# The development of spatial behaviour and the hippocampal neural representation of space

**DOI:** 10.1098/rstb.2013.0409

**Published:** 2014-02-05

**Authors:** Thomas J. Wills, Laurenz Muessig, Francesca Cacucci

**Affiliations:** 1 Department of Cell and Developmental Biology, University College London, London WC1E 6BT, UK; 2 Department of Neuroscience, Physiology and Pharmacology, University College London, London WC1E 6BT, UK

**Keywords:** development, hippocampus, navigation, spatial, place cell, grid cell

## Abstract

The role of the hippocampal formation in spatial cognition is thought to be supported by distinct classes of neurons whose firing is tuned to an organism's position and orientation in space. In this article, we review recent research focused on how and when this neural representation of space emerges during development: each class of spatially tuned neurons appears at a different age, and matures at a different rate, but all the main spatial responses tested so far are present by three weeks of age in the rat. We also summarize the development of spatial behaviour in the rat, describing how active exploration of space emerges during the third week of life, the first evidence of learning in formal tests of hippocampus-dependent spatial cognition is observed in the fourth week, whereas fully adult-like spatial cognitive abilities require another few weeks to be achieved. We argue that the development of spatially tuned neurons needs to be considered within the context of the development of spatial behaviour in order to achieve an integrated understanding of the emergence of hippocampal function and spatial cognition.

## 1. Introduction

The hippocampal formation plays a fundamental role in spatial cognition and navigation, across the whole vertebrate group [1–6]. This role is thought to be supported, at the neural level, by the presence of several different classes of neurons whose firing is tuned to an animal's position and orientation in space. Some of the principal spatial cell responses are place cells, which fire in a unique position in an environment and encode the animal's current location [7]; head direction (HD) cells [8], which encode the heading direction of the animal; grid cells, which fire in several locations in an environment, laid out in a hexagonal grid, and may encode distance travelled [9]; boundary vector/border cells, which respond to boundaries of the environment and may represent fundamental inputs to place and grid cells [10,11]. Spatially tuned neurons have been most intensively studied in rodents (laboratory rats and mice in particular), but similar neuronal activity has been described in a range of mammalian species, as well as in other vertebrates [12–19].

An intense and long-standing research effort has addressed the function of this neural representation of space, and how it underpins spatial cognition in the adult rodent [20,21]. More recently, several groups have also begun to approach the question of how and when the hippocampal neural representation of space emerges during development [22–25] (see also [26] for earlier work). The results of this work show that the different types of spatially tuned neurons emerge at different times during development, within the first three weeks of the rat's life, and follow different developmental programmes thereafter [22–25]. So far, this research has not only provided insights into the developmental mechanisms responsible for the emergence of the neural map of space, but, crucially, also into how this might function in the adult.

The purpose of this review is to summarize what is known of the development of spatial behaviour in the laboratory rat (*Rattus norvegius*), encompassing both the emergence of spontaneously expressed spatial behaviour, as well as formal testing of spatial cognition in tasks known to be dependent on an intact hippocampus in the adult. In order to provide a meaningful context to the emergence of spatial cognition, we will also summarize the most important landmarks in the sensory and motor development of the rat, focusing on those aspects that are thought to be necessary to express spatial learning and behaviour.

We will compare the development of spatial behaviour with the emergence of spatially tuned responses in the hippocampal formation, with the view of addressing two questions. First, does the relative timing of the emergence of spatial behaviour and spatial firing allow us to infer the relative roles of sensory experience and endogenous factors in their development? The original proposal of the hippocampus as a cognitive mapping system [1] argued that the neural representation of allocentric space supported by the hippocampus was a Kantian *synthetic a priori* system, meaning that it did not require empirical experience for its construction, or for its validation. Rather, the cognitive map was predicted to be independent of prior experience of spatial relations in the world, and form a scaffold for the coherent organization of those experiences. In this sense, the cognitive map theory predicted that the ontogenetic development of spatially tuned neurons should be independent of experience of space [22].

The second question that will be addressed in this review relates to the functional link between the hippocampal neural representation of space and spatial cognition. In the adult rat, the firing of spatially tuned neurons has been shown to correlate with the accuracy of behaviour in tests of spatial cognition [27–30]. Do ontogenetic studies allow us to look for such functional relationships, that is, does the developmental timeline of spatially modulated firing indicate that it is a prerequisite for the expression of spatial behaviour?

More generally, establishing the relative developmental timelines of spatial behaviour and spatially modulated firing is an important step in understanding the interaction of the different factors (e.g. molecular cues, intrinsic neural activity and environmental experience) that are involved in the development of neural systems [31–34]. The development of the hippocampus at the molecular, cellular and physiological level will not be discussed in this review, but the reader is directed towards other recent reviews that cover these areas [35–37].

## 2. The development of sensory-motor systems in the rat

### (a) The development of sensory systems

Like humans, rats are altricial animals, and newborn rat pups have limited sensory perception. Sensory development in the rat follows the general mammalian blueprint of sensory ontogenesis, with the vestibular and olfactory functions being the first to emerge (around birth) followed by tactile, auditory and visual function, respectively [38].

At birth, rats display a rudimentary righting reflex [39], possibly reflecting the functioning of an immature vestibular system. Recordings of peripheral or central vestibular neurons in anaesthetized animals show that weak neural responses to rotation exist as early as postnatal day 1–2 (P1–2), which become largely adult-like by P8 [40–42]. The behavioural development of vestibular responses has not been extensively tested in rats, but it is known that pups can right in mid-air during a fall from P17 onwards [39]. In the mouse, the optokinetic and vestibulo-ocular reflexes are essentially adult-like at P21 [43]. The vestibular system is therefore thought to be present in immature form from the first few days of life and develop substantially during the first three weeks of the rat's life.

The other precocious sensory modality in the rat is olfaction: rats demonstrate a preference for their mother's odour at 2 days of age [44]. Rudimentary sniffing behaviour can be observed at P4, but it is not until P10–11 that the typical adult sniffing behaviour (in combination with head pointing) emerges, and by P15 rat pups systematically sniff at everything in their home cage [39,45].

Tactile exploration in rats is thought to be principally mediated by active whisking (high-frequency-directed movements of their facial macrovibrissae) [46]. Movements of the whiskers are first observed at P4, but adult-like whisking (repeated cycles of retraction and protraction) does not emerge until P10–13, after which the frequency and amplitude of whisking movements continue to develop until around P21 [47–49]. However, even before P4, passive movement of the whiskers can induce activity in rat pups, and whisker clipping disrupts suckling and huddling behaviours at P4–5 [50].

The auditory system of infant rats begins to function at P8–9, at which age cochlear microphonic potentials (reflecting hair cell stimulation) can be observed. Action potential responses (reflecting action potentials in the auditory nerve) first occur at P11–12 [51]. The auditory meati of rat pups open at P11–13, and it is around that time that rat pups start showing clear orienting towards an auditory stimulus [45,52].

Vision is the last sensory system to emerge, with the eyes opening at P13–15 [39,45,52–54]. The optics of the eye are not clear until P19 [53], and electrophysiological recordings from primary visual cortex reveal very immature responses to a series of bars and gratings at P17–19 (reduced acuity, larger receptive fields and insensitivity to orientation or movement direction) [53]. However, a more recent study [54] reported that neuronal preference for the orientation and spatial frequency of a grating is largely adult-like at P16, though the contrast threshold is lower than that in adults.

The precocious sensory modalities (vestibular, olfaction, tactile sensation) are therefore probably generally adult-like by three weeks of age, in clear contrast to the visual system, which is functional, but remains largely immature at this age.

### (b) The development of motor skills

During the first week of life, relatively simple motor behaviours prevail. At P0, pups can invert their posture, either to right themselves or to feed from their mother, and move a small distance along the mother's ventrum to arrive at a nipple [55]. Altman & Sudarshan [39] performed an extensive series of tests of the development of motor skills in Wistar rat pups, testing animals every day between P1 and P21. It is important to note that these tests were conducted in an open field, where pups were individually tested while away from their mother and littermates. The first organized locomotor behaviour observed under these conditions was ‘pivoting’, turning on the spot that is driven by movement of the forelimbs, whereas the hindlimbs remain immobile. Pivoting began at P3, peaked at P7 and diminished thereafter. Pups first became capable of translational movement at around one week of age, although such movements were inefficient (termed ‘crawling’), with the hindlimbs mostly dragged along, rather than contributing to movement. Full quadrupedal walking, including proper, coordinated use of hindlimbs, emerged from P14 onwards. Rats did not actively move around in the open field until P9, started to travel short distances between P10 and P14, and the distance covered increased abruptly from P15 onwards. By P21, pups were capable of a large range of complex motor skills, including rope climbing, traversing a narrow raised walkway and jumping down a vertical drop.

The results of this study suggest that, similar to the development of sensory systems, the development of the species-specific motor repertoire of the rat takes place across the first three weeks of postnatal life.

## 3. The development of spatial behaviour

**Figure 1.**
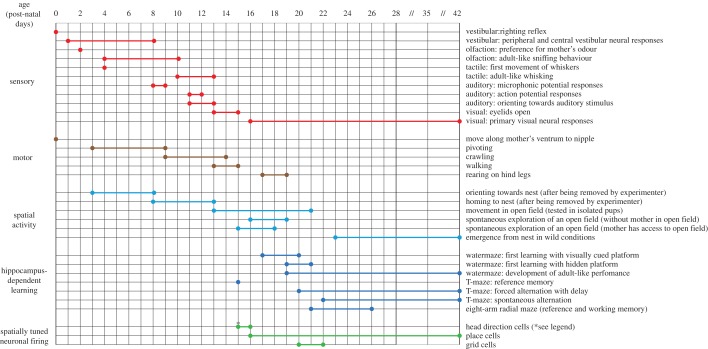
Summary timeline of sensory, motor and spatial cognition development in the rat. Each horizontal line represents the development of a particular spatial behaviour, motor ability or sensory function. Vertical lines indicate age between postnatal days 0 and 42 (P0–P42); note the compressed horizontal scale between P28 and P42. Bold horizontal lines (ending in circles) represent the beginning and the end of the developmental emergence of each trait. Single circles indicate the existence of a spatial behaviour, motor ability or sensory function at a given age. Different colours indicate different developmental trends: red, sensory; brown, motor; light blue, spontaneous spatial activity; dark blue, hippocampus-dependent spatial learning; green, spatially tuned neuronal firing in the hippocampal formation. Asterisk denotes that the age of the earliest emergence of head direction cells is not yet known. (Online version in colour.)

In this review, we will adopt a definition of spatial behaviour that encompasses all spontaneously expressed movements through space (§2*a*), as well as the ability to solve formal tasks of spatial learning and navigation, that are dependent on an intact hippocampus in the adult rat (§2*b*). The data from the studies discussed below are also summarized in figure 1.

### (a) The emergence of spontaneous movements through space

During the first two weeks of life, rats spend most of the time in their nest, with their mother and littermates (see below, ‘Activity in the nest’). The emergence of spontaneous movements outside the nest occurs, in laboratory animals, sometime within the third week of life.

#### (i) Homing

One of the earliest expressions of spatial behaviour is the ability of pups to return to the nest if separated from their mother and littermates by the experimenter. Altman & Sudarshan [39] tested when pups could return to the nest group within 3 min when separated by a 20 cm direct line: at P7, none could achieve this; by P10, the success rate was approximately 50%; by P13, 100% of pups could return to the nest. However, this task was probably testing the development of crawling ability, as well as the ability to locate the nest: almost 100% of pups could correctly orient towards the nest by P8. In a more formal homing test, Bulut & Altman [56] probed whether rats could learn to discriminate between two adjacent doors in order to return to the nest. Training started at P6, but no pup performed successfully on this task until P13. In summary, rat pups develop the ability to successfully orient towards the nest (probably using olfactory and auditory cues) and locomote towards it during their second week of life.

#### (ii) Exploration and leaving the nest

The earliest studies of rat development noted the striking prominence of exploration and curiosity in immature rats [45,57,58]. In adult rats, exposure to novel environments prompts a well-defined exploratory response [1,59,60] which depends upon an intact hippocampus: hippocampal-lesioned animals are hyperactive, but do not systematically explore their environment, and show less habituation after repeated exposures to an environment [1]. Pinpointing the emergence of spontaneous exploration may therefore inform us about the development of hippocampal function. It will also help to define at which age rats experience large-scale space during the course of normal development: as described below, the development of locomotor skills, when tested in isolation [39], does not necessarily map onto the normal emergence of spontaneous exploratory behaviour.

When isolated pups are placed in an environment, they will walk from around two weeks of age [39,61]. If isolated pups are left for longer in the testing arena (25–120 min), then P20–21 pups will habituate to the environment, as shown by decreasing activity levels, but P15 pups will continue to move at similar levels throughout the whole testing period (reminiscent of hippocampal-lesioned animals [1]), leading to an overall peak in motor activity at P15 [62,63], see also [22,23]. This behaviour is dramatically changed, however, by the presence of a conspecific in the open field. The heightened activity levels of P15 pups can be inhibited by the presence of littermates, an anaesthetized lactating female, or even an anaesthetized male [64], suggesting that heightened open field activity around P15 may reflect a response to social isolation, rather than spontaneous exploration of the environment. In keeping with this interpretation, ultrasonic vocalization (USV) distress calls, which are an anxiety-based response to social isolation in rat pups, peak during the second week of life, are still observed at P14, but are much reduced by P18–19 [65–68].

Nadel *et al.* [69] also found a dissociation between the ontogenetic profile of motor activity, as tested in a running wheel, and that of active exploration, as tested by placing single rat pups in an open arena containing several objects. A peak in motor output in the running wheel occurred around P16–17, contrasting with the emergence of significant exploration of the arena, which appeared between P17 and P25 (median P21). Perhaps the most intriguing observation in this study was that the onset of active exploration was abrupt within each pup, occurring almost overnight, in an all-or-none fashion.

Consistent with this latter study, the age at which pups will *spontaneously* leave the confines of the nest to explore the surrounding environment is towards the end of the third week of life. Pups reared with free access to an eight-arm maze (while the mother was confined to the nest-box) did not make any forays into the maze between P13 and P15, a small amount of activity was seen on P16, and activity levels rose steeply thereafter [70]. Similarly, pups placed on an open platform with their littermates (but without their mother) make only very short forays away from the huddle between P16 and P18, and the distance of these forays increases from P19 onwards [71]. Likewise, pups prefer to explore the novel side of a box only from P19 onwards [72]. Rearing on the hind legs, an important behavioural marker of novelty-induced exploration [73], first emerges at around P17, and increases rapidly from P19 onwards [39].

The presence of the mother outside the nest is also an important stimulus that can increase levels of nest egression. At P19, pups make more spontaneous forays from a nest-box into an open field if the mother is present there [74]. Bearing this in mind, probably the most ethologically valid approach is to determine the emergence of exploration when both mother and pups have free access to an open field. When this is the case, pups tend to spend a small amount of time (less than 10%) in the open field between P15 and P17, and show a sudden increase to approximately 30% of time spent in the open field at P18 [75]. This study also found that the developmental emergence of nest egression is modulated by the ambient temperature, presumably reflecting the temperature regulation function of huddling behaviour (see §3*a*(iv)). The results described above refer to pups raised at 21°C; pups raised at 30°C spend more than 50% of their time in the open field at P16, whereas at 18°C, less than 10% of time is spent in the open field even at P20. For all temperatures, the amount of time spent in the open field is approximately zero at P14, setting a well-defined lower limit to the earliest age of exploration.

Full independence from the mother occurs much later in development: although weaning is commonly induced in laboratory rats at P21, rats left with their mother will wean at around P35 [76]. Furthermore, the exploration of an open field and of objects has been shown to continue to mature between P30 and P90 [77,78].

In summary, the onset of spontaneous exploration outside the confines of the nest happens during a narrow time window, centred around the end of the third week of life in the rat. Moreover, there are indications that the transition to exploratory behaviour is abrupt in each animal, suggesting that this transition might reflect specific, as yet unidentified, neural changes in the hippocampal circuitry.

#### (iii) Egression from the nest and the role of path integration

Path integration describes the ability of an animal to home back in a straight trajectory to a starting position (e.g. nest) after an excursion, calculating the distance and direction traversed on the outbound journey on the basis of internally generated cues (e.g. vestibular cues, motor efference copy or proprioception) [79,80]. Path integration is thought to be one of the two principal processes (along with information acquired from the location of external landmarks) in the generation of a neural map of space [81,82]; it is generally accepted that path integrative behaviour is disrupted by lesions to areas containing spatially tuned neurons [83–85], but see also [86]. It is therefore of interest to study the ontogenetic time course of path integration. Loewen *et al.* [71] reported that the exploratory behaviour of rat pups was consistent with an ability to path integrate, even at very early ages (P16 onwards). On a 1.5 m diameter platform, return trips to the huddle were always shorter than outwards forays, even in the dark and upon removal of the olfactory and auditory cues provided by the presence of littermates.

#### (iv) Activity within the nest

Although egression from the nest marks the first movements in large-scale space, pups are far from immobile within the nest environment [55,87–89], and their activity patterns during this time may well constitute a critical experience of movement and orientation in space. Before active exploration outside the nest begins, the predominant environment of the pup is the ‘huddle’, an aggregation of littermates within which each individual pup will attempt to move to the centre, and be pushed to the periphery by littermates attempting to do likewise [87]. The purpose of such aggregation behaviour is probably thermoregulation: increasing the ambient temperature can change the ‘convective flow’ of pups within the huddle from inwards (as described above) to outwards [87]. Huddling behaviour gradually breaks down as pups become more active, sometime during the third week of life [71].

Another manner in which rat pups sample space within the nest are movements with respect to their mother [55,88–90]. Rat pups display orienting towards the mother even at P1–3 [55,89], move around the body of the mother [88] and shift between nipples while suckling from P10 onwards [91]. Cramer *et al.* [91] reported that restricting nipple shifting between P5 and P24, by surgical removal of nipples, led to learning deficits in an eight-arm radial maze (see below) at P25, and suggested that nipple shifting may constitute experience of space and sequence learning. The specificity of this deficit to nipple shifting should probably be treated with caution, however, as surgical removal of the nipples may have altered the pup–mother interaction, which could have also affected the normal course of development in a more general way. In particular, the hippocampus has an important role in the stress response in the adult, and stress modulates the efficacy of hippocampal learning and synaptic plasticity [92]. It has been shown that stress during development, including variations in maternal care, can alter the hippocampal response to stress and hippocampal function in spatial cognition, later in adult life [93,94].

#### (v) Development in a non-laboratory environment

Comparatively, little is known about the behaviour of Norway rats in the wild. An ethological perspective is important for ontogenetic studies, as the developmental programme of behaviour may be evolutionarily adapted to an environment dissimilar to laboratory rearing conditions: knowing the ‘natural’ environment can help understand the interaction between the individual animal and its environment during development [95].

Calhoun [96] raised a colony of wild-born Norway rats in an enclosed outdoor pen approximately 1000 m^2^ in area. The development of three litters was described in detail in this study: in all cases, the first age at which nest egression was observed was considerably later (P23–P40) than described in the laboratory studies above (but note the late egression from the nest in cold conditions found in [75]). Note that two of these three examples occurred within the first months of the existence of the colony, and therefore would not have been affected by the high mortality and low reproductive success that affected the colony towards the end of the study. It seems that emergence from the nest therefore occurs at younger ages in a laboratory setting compared with wild or semi-wild conditions. Thus, the end of the third week of life might be considered a lower bound when trying to identify the hippocampal processes that might underlie the onset of active exploratory behaviour in the rat.

In Calhoun's study [96], nest chambers were often contained within extensive, topologically complex burrow systems, though mothers also made nests in ‘harbourage boxes’ at surface level, provided by the experimenters. In burrow systems, the modal distance from a nest chamber to the nearest exit was approximately 0.5 m. Some studies have found that the burrows of domesticated laboratory rats are considerably more basic than those of wild Norway rats [97,98], though Boice [99] found no difference between the burrows of wild rats and domesticated albino rats raised in an outdoor pen. In general, a nest chamber, connected to the external world by a short tunnel, would appear to define the geometry of the space into which a pup would be born, in non-laboratory conditions.

### (b) The development of hippocampus-dependent spatial learning and memory

In this section, we review the development of spatial learning and memory, as tested formally by behavioural tasks which require intact hippocampal function in the adult rat. We will not discuss the development of other forms of associative learning (for example, the development of conditioning): for this work, the reader is directed to the review contained within [35].

#### (i) Water maze

The Morris water maze task, in which rats are required to learn the location of a platform in a circular swimming pool [100], is one of the most widely used behavioural paradigms in the assessment of spatial learning and hippocampal function. In the ‘place navigation’ version of this task, the platform is hidden below the water surface, meaning that rats cannot rely on local cues when finding the platform. In adult rats, lesions [2] as well as pharmacological inactivation [101] of the hippocampus abolish the ability to display such place navigation. If a salient visual cue is placed near the platform (the ‘cued’ version of the task), then the ability to find the platform is independent of hippocampal function [2], and is therefore thought to reflect the use of a different navigational system by the animal.

The first study of the ontogeny of place navigation in the water maze found that although adult-like learning (including multiple crosses through the platform site when the platform was removed) was not observed until P42, there was evidence of learning, reflected by reduced latencies to find the platform, even at P21 [102]. Several studies have since attempted to determine the first onset of place learning in the water maze. Rudy *et al.* [103] showed that rats trained on days P18 and P19 demonstrated reduced latencies to find the platform at P19, but no preference to visit the platform location when it was removed from the pool. Rats trained on P20 and P21, however, showed latencies which reduced within the day, on both P20 and P21, and a preference for the platform location at P21. Brown & Whishaw [104] confined training to 1 day only, and took precautions to ensure that training in the water did not lead to a drop in body temperature. In this case, evidence of place learning was observed at P19 and P20, but not at P18. Akers & Hamilton [105] also confined training to 1 day, finding that the first evidence of reduced latencies can be observed at P20, but a clear preference for the platform quadrant in the no platform probe appears to develop later, between P20 and P24. Finally, it should be noted that weanling pups (P20–P28) show increased learning when the maze is scaled down to an appropriate size [102,106], and in a 40 cm diameter maze, rats trained between P17 and P19 showed evidence of place learning at P19, when compared with naive P19 rats.

In summary, no study has found evidence of place learning earlier than P19, and most studies agree that this ability is present by P21. It should be reiterated, however, that fully adult performance does not emerge until much later [102].

Most studies agree that the visually cued version of the task can be solved 1–2 days earlier during development than the hidden platform version [102,103,105], with the earliest evidence of learning (assessed by reduced escape latencies) being observed at P17 [103,105,107]. (Though note that one study failed to find evidence of this, with both place and visually cued learning emerging in parallel at P19 [104]). The earlier emergence of learning on the visually cued water maze has been interpreted as evidence that the different brain systems involved in place- and landmark-guided navigation [1,2] are maturing at different times, and could also be taken as evidence that the limiting factor in the developmental emergence of place navigation is the development of hippocampal function, rather than immature sensory or motor systems (as the visually cued water maze requires rats to visually locate the platform and swim to it). However, some caution should be applied when considering this interpretation: visual acuity is still very immature around three weeks of age, and experimentally reduced visual acuity in the adult rat leads to deficits in the place, but not the cued version of the water maze [108]. The developmental lag between place- and cued- navigation might simply reflect the later age at which visual acuity is sufficient for proper perception of the extra-maze cues. Consistent with this hypothesis, P19 rats show reduced escape latencies and increased preference for the platform location when the platform is positioned closer to the distal cues, or the number of distal cues is increased [109,110]. Using a dry-land variant of the water maze (requiring an escape though one of a series of holes in an open arena), Rossier & Schenk [111] showed that rats gave more emphasis to local olfactory cues than distal visual cues until after P48. Interestingly, if rats were trained with both visual and olfactory cues, and then the olfactory cues were removed, P48 rats could rely on visual cues on their own, but P24 rats could not, suggesting a late development of multi-sensory integration in the hippocampus.

To further test the role of extra-maze cues in the water maze, Hamilton *et al.* [112] introduced a variant of the task in which the pool is shifted relative to the laboratory and distal extra-maze cues. In this situation, adult rats have a strong tendency to swim towards the platform position as defined by the pool, rather than that defined by the extra-maze cues. The authors [112] interpret this as evidence of a navigation strategy that primarily uses a directional bearing to the platform (termed ‘directional’ navigation), but an alternative interpretation is that the boundaries of the pool play a primary role in defining platform position, and the extra-maze directional reference frame serves to disambiguate positions within the circularly symmetric pool. Adult rats can learn to navigate to an absolute position in the extra-maze reference frame, ignoring the pool boundaries (termed ‘place’ navigation), if trained on a procedure that explicitly dissociates the platform position from the pool boundaries, and if the pool boundary is eliminated as a sensory cue as much as possible (for example, by filling the pool completely with water) [113]. Akers *et al.* [114,115] conducted a series of studies investigating when ‘directional’ and ‘place’ navigation emerged during development, and showed that ‘directional’ navigation emerged at approximately P20, whereas ‘place’ navigation emerged later, at around P26. One possible caveat is that this may, again, reflect protracted visual sensory development: it is possible that using several distal cues to precisely triangulate a position requires a higher visual acuity than using these cues only as a directional fix. Interestingly, animals as young as P17, trained using a visually cued platform, will show disrupted performance if the cued platform moves to a new position relative to the pool boundaries [107]. This demonstrates that a ‘directional’ learning strategy (i.e. the use of the pool boundaries as a spatial cue and an extra-maze directional fix) is present in animals as young as P17, and furthermore, that this is not overshadowed by the presence of a visual landmark during learning.

Brown & Kraemer [116] tested the development of long-term memory retention in the water maze, and found that a 3- or 7-day delay between learning and testing resulted in worse performance, compared with no delay, at P20 and P34, whereas adults performed equally well in all conditions. Likewise, Spreng *et al.* [117] found that a spaced training protocol does not aid long-term retention at P33, unlike in adults. These studies suggest a protracted development for the long-term retention of spatial memory in the hippocampus, although Carman *et al.* [118] found that early experience in the water maze can influence learning at a later date: pups trained at P17–19 performed better than naive pups at P26, after a limited ‘reinstatement’ training session.

#### (ii) T-maze

The T-maze can be run using various protocols, including ‘reference memory’, in which the goal location is constant, and ‘delayed forced alternation’, in which the rat is first forced into one goal arm (by blocking access to the other), then subsequently required to choose the opposite arm from a free choice, possibly after a variable delay [119]. Green & Stanton [120] investigated the ontogeny of learning on both the reference memory and delayed forced alternation protocols. It was found that pups as young as P15 can learn the reference memory task, but if forced alternation and a 15 s delay are introduced, then the behaviour of P15 pups falls to chance levels. P21 and P25 animals can learn the delayed forced alternation task after 20–30 trials, however, even at P33 rats are still more likely to make errors than adult animals [121]. Freeman & Stanton [122] found that fornix lesions at P10 prevented rats from learning the delayed forced alternation T-maze (when tested at P23), but not the reference memory T-maze, suggesting that the early emergence of the latter is based on non-hippocampal neural systems.

When presented with a free choice between two maze arms, adult rats will naturally tend to alternate between them, a behaviour that is generally abolished by hippocampal lesions [1]. Kirkby [123] reported a gradual increase in rates of spontaneous alternation between P20 and P80, with P20 animals performing at chance levels. Douglas *et al.* [124] investigated the emergence of this behaviour in more detail, showing that most animals reach a criterion (75% alternation across 20 consecutive trials) between P23 and P33, but a small fraction of animals reaches this criterion only between P61 and P65. More remarkably, within individual animals, the transition between performing at or around chance levels to reaching the criterion occurs within a very short period (few days), an effect that is obscured when one only looks at the average performance across animals. Echoing the slow maturation of long-term memory in the water maze, Bronstein *et al*. [125] reported adult-like performance in P30 rats when testing spontaneous alternation across a 15 s delay, but a reduction in performance to chance levels when using a 1 h delay.

#### (iii) Radial arm maze

In the simplest version of the radial arm maze, rats need to collect the reward from all arms of the maze, learning not to return to previously visited arms [126]: the acquisition of this task requires an intact hippocampus [127]. Rauch & Raskin [128] trained one group of animals on this task repeatedly from P16 until P25, and found that rats could not complete the task (defined as visiting all eight arms in 15 min) until P21, and the success rate at P21 (defined as the number of different arms entered in the first eight choices) was above chance. A different group of animals, trained for one developmental day only (age range P21–P25), could complete the task by P22, and showed a sharp increase in performance on P23 (when judged by a reduction in the number of repeat arm entries until all eight pellets were eaten). When animals were tested on a mixed reference/working memory paradigm (the same four arms out of the eight always baited) [126], both reference and working memory scores were above chance from P21 onwards. The ability to solve this version of the radial arm maze therefore emerges at approximately three weeks of age.

In summary, formal testing of spatial cognition in rats shows that the first evidence of hippocampus-dependent navigation emerges around three weeks of age (see figure 1 for summary). However, the full complement of adult abilities unfolds over a longer timescale, achieving maturity between six and eight weeks of life.

## 4. The development of the neural map of space

Spatially responsive neurons in the hippocampal formation are thought to represent the neural underpinnings of cognitive maps, i.e. mental representations of the relative location of objects and landmarks in space that can be used for navigation [1,129]. In the following sections, we will review the recent work that has begun to uncover when the spatially modulated firing of these cells emerges during development [22–24], and, whenever possible, highlight potential functional links between the emergence of spatial signalling in the hippocampus and spatial behaviour in immature rats.

### (a) Head direction cells

HD cells encode the current heading direction of the animal. Each HD cell fires whenever an animal points its head in a specific allocentric direction, as defined by both exteroceptive (e.g. external sensory landmarks: visual, auditory, olfactory) and interoceptive (e.g. vestibular, self-motion signals, motor efference copy, optic flow information) cues [130].

**Figure 2.**
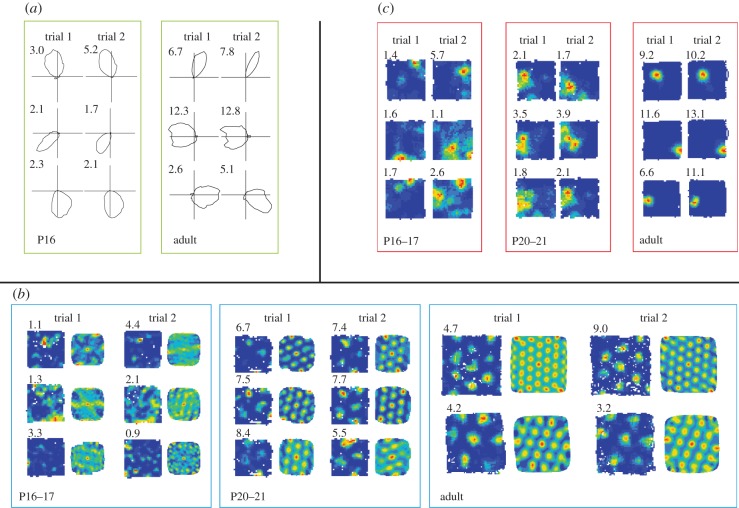
The development of spatially responsive neurons in the hippocampal formation. (*a*) Three HD cells recorded from medial entorhinal cortex at P16 (left), and from adults (right). Each polar plot represents the firing rate (action potentials per seconds of dwell time) for each directional heading, the peak firing rate is shown at the top-left corner. Each row represents one cell, each column a separate recording trial (separated by 15 min). Note the similarity between P16 and adult HD cells, in terms of directional selectivity and the stability of the preferred direction of firing. (*b*) Medial entorhinal cortex cells recorded at P16–17, P20–21 and from adult rats. Each row shows a cell, each pair of columns a trial. For each trial, the left column shows a false-colour firing rate map, the right column a spatial auto-correlogram of the firing rate map, which highlights hexagonal grid structure. Peak rate (action potentials per seconds of dwell time) is shown in the top-left corner of the rate map. Note the absence of stable and regularly symmetrical grid firing fields at P16–17. Note also that adult recording arenas are larger than those for immature rats; rate maps are shown to scale. (*c*) Three place cells recorded from CA1 at P16–17, P20–21 and from adult rats. Each row shows a cell, each column a trial. Peak rate (action potentials per seconds of dwell time) is shown in the top-left corner of the rate map. Note the gradual improvement in the specificity of spatial tuning, and the stability of the place field position. (Online version in colour.)

Intriguingly, of those spatially responsive neurons studied so far (HD, place and grid cells), HD cells are the first to emerge during development. Adult-like HD responses, which are stable both within and across two recording sessions, separated by approximately 15 min, can be recorded from both the medial entorhinal cortex and the dorsal presubiculum, in rat pups that are at least 15- to 16-days old [22,23,131,132] (figure 2). This is an age preceding significant active exploration (see §3*a*(ii)), suggesting that the HD circuit might be wired in the absence of active exploratory experience, and that its wiring might rather rely on endogenous mechanisms. Consistent with this interpretation, the amount of experience in the recording environment does not correlate with the quality of HD firing in these immature pups [22]. The early maturation of the HD system might, more specifically, reflect the early maturation of the vestibular system (see §2(a)), with which the HD circuit is intimately connected [130]. Adult-like HD responses can be recorded at an age when place responses are still mostly immature (see below) and when no stable grid cell responses can be detected (see below), suggesting that HD responses might be the ‘primary’ spatial signal, and be independent of other spatial neurons for their function or development. This hypothesis is consistent with observations that lesions to [133], or temporary inactivation of [134] the hippocampus (where place cells are located) have little impact on HD cell firing in adults.

### (b) Grid cells

Grid cells fire in multiple locations arranged in a hexagonally symmetrical grid, and may encode for the distance travelled by the animal [9]. They can be recorded from the whole of the parahippocampal region (medial entorhinal cortex, pre- and parasubiculum) [135,136].

Two studies have so far traced the emergence of this spatial signal and demonstrated that the first stable, adult-like grid responses can be recorded from the medial entorhinal cortex not earlier than P20 [22,23] (figure 2). From thereafter, one can observe a swift improvement in both stability and spatial quality (as measured by gridness) within the next few days of life. Grid cell responses thus emerge about a week later than fully mature HD cells and the earliest place cells (see below). Their emergence roughly corresponds with the age at which weaning is induced in laboratory animals (P21) and pups are therefore required to start an independent life, as well as the age at which hippocampus-dependent behaviours start to emerge (see §3*b*).

Some of the distinctive properties of the grid cell network are already present as soon as these signals can be detected. Adult grid cells are arranged in functional ‘modules’ [9,137,138]: within each module, all grid cells share the same wavelength and orientation, and the relative spatial phases of the grid fields remains fixed, even though the absolute position of their firing peaks (phase) can change [139,140]. This coherent network structure emerges concurrently with the first stable grid cells, from P20 onwards [25]. Other adult-like characteristics of medial entorhinal cortex firing, such as the existence of ‘conjunctive’ (combined grid and HD tuning) cells, and speed-modulation of grid cell firing [135], also emerge at around P20 [25]. These data suggest that local networks of stable grid cells emerge as coherent units, relatively abruptly during development.

### (c) Place cells

Place cells fire whenever an animal occupies a specific location in its environment (the ‘place field’ of the cell) and, as such, are thought to encode the current location of the animal [1,7].

Several studies have tracked the development of this spatial cell type in the rat [22–24,26]. At the earliest ages sampled (P16), place cells appeared to be on the whole immature, with most place fields displaying lower spatial stability than those of adult rats, both within and across repeated exposures to the recording environment [22,23]. The specificity of spatial tuning (as measured by spatial information) was also significantly lower in the youngest pups, compared with adults, in [22] (though not in [23]). For examples, see figure 2. Interestingly, both these parameters seem to improve monotonically during the following two weeks of the rat's life, with one report suggesting that the place cell network might approach maturity at around P45 [24]. This gradual improvement in place cell responses is very much at odds with the relatively sudden appearance and maturation of grid cell firing, and these distinct ontogenetic modes may reflect the different developmental mechanisms underpinning their emergence.

This hypothesis is further strengthened by the observation that there is a large variability in the quality of place responses recorded from rat pups at any given age [22–24]. Even at P16 (the earliest age sampled so far), a subset of place cells display adult-like stability and spatial quality [22,23], suggesting that the development of place responses is not taking place at a network level, but at the single pyramidal cell level. This variability might reflect the nature of the inputs each pyramidal cell receives during development, or factors endogenous to each cell. This is in marked contrast with HD and grid cell development, where there are strong indications that development takes place at the network level [22,23,25]. It is also important to note here that as for HD and grid cells, the improvement in the spatial quality of place cell responses does not correlate with experience in the recording environment [22].

The emergence of place cells several days earlier during development than grid cells was an important piece of evidence against the previously commonly accepted model that grid cells formed the principal input to place cells [81,82]. One alternative possibility is that the earliest building blocks of place cell firing might rather be boundary vector cells [11], consistent with a long-standing theoretical model [141] predicting that place cell firing fields are based on inputs from boundary-responsive cells. Preliminary evidence from recording in pre-weanling rats indicates that boundary-responsive cells can be recorded from at least P17 onwards [142,143], and, furthermore, that boundaries may form an important functional input to place cells in pre-weanling rats [143].

## 5. Conclusion and open questions

In reviewing the emergence of spatial behaviour and spatially modulated neural firing in the hippocampal formation of the rat, we endeavoured to provide an answer to two fundamental questions: (i) what are the relative roles of sensory experience and endogenous mechanisms in shaping the development of hippocampal spatial networks; (ii) how does the development of spatial responses at the neural level interact with the development of navigational ability? In this concluding section, we will attempt to provide tentative answers to these questions and highlight the gaps of knowledge that need to be addressed by future research.

### (a) The role of experience in the development of the neural representation of space

One of the most striking findings to emerge from studying the development of spatially responsive neurons is the precocious development of the HD system [22,23], with adult-like responses emerging before the onset of active exploration [70,71,75]. The neural representation of direction in the hippocampal formation (and other brain regions [130]) may therefore qualify as a Kantian synthetic *a priori* system, insofar as its construction during development may not require experience of exploring large-scale space. However, while P15–16 is certainly before the emergence of extensive exploration [69–71,74,75], it is also on the cusp of when initial, tentative forays outside the nest begin [70,75]. In this regard, it is interesting to note that preliminary evidence pinpoints the emergence of HD cells to P14 [132], an age before any exploration outside the nest is observed at all [70,75]. A further possible caveat is that active exploration of space outside the nest is only one type of experience that occurs during development, and the crawling, turning and diving motions that rats experience within the huddle [87] may be necessary to set up the vestibular and motor inputs into the HD system [130]. On the other hand, HD cells in adults are noted for their dependence on distal visual cues for stability [144], and how the network would function in the confined space of the huddle, in functionally blind animals [53], is not at all clear.

Most network models of HD cells centre around a common type of architecture, termed a ‘continuous attractor’ network [145,146]. The only network model of HD cells to directly address their development [147] proposes that a continuous attractor network is created by experience-dependent learning, with input from a stable visual landmark combining with vestibular input to shape the developing network. To date, no formal neural network model exists explaining how the intricate set of connections necessary for a continuous attractor could develop independently of sensory experience.

Both grid cells, and a fully mature place cell network [22–24], emerge at a point in development after extensive exploration of space; therefore experience of exploration could be a necessary part of their normal development. Network models of grid cell development using experience-dependent learning have been proposed [148–150], though it may be important that, in all these models, learning depends on input from pre-existing spatially tuned neurons, leaving the ontogeny of this ‘teaching’ signal still unexplained. It is also important to note that Wills *et al.* [22] found no correlation between experience of the recording arena and the maturity of grid or place cells, suggesting that there was no role for learning to associate spatial firing with specific places, though this does not rule out a role for the experience of space *per se*.

It may also be useful to consider the natural environment in which rats would develop outside of the laboratory. If the developmental programme of rat pups has been adapted by natural selection to an environment consisting of restricted nest chamber linked to a narrow tunnel [96,99], this may dictate how the neural representation of space develops. In particular, it may explain why neural representations of direction [22,23] and boundaries [142,143] (the defining spatial features of a tunnel system) emerge earlier than those spatial responses (for example, grid cells) that map open spaces [22–24], and may also predict differential roles of experience on the development of these cell types.

### (b) The role of the neural representation of space in the development of spatial behaviour

The appearance and rapid maturation of the grid cell network seems to mark the age at which there is a transition to a hippocampus-dependent navigational system, at around three weeks of age [22,23,102–105,120,128], possibly underlying the functional coming ‘online’ of the hippocampal formation. One problem with this interpretation is that place cells in CA1, generally thought of as one of the major output structures of the hippocampal formation [151], remain, on average, rather immature at three weeks of age, and furthermore show no abrupt developmental change at this time. If grid cells do represent the critical missing component necessary for adult-like hippocampal function, then the spatial information they convey is either being transmitted via different anatomical pathways, or is encoded in the activity of CA1 cells in a way that is, so far, not apparent. It should also be noted that, to date, no interventional or even correlational studies have been conducted to assess whether the emergence of grid cells and hippocampus-dependent behaviours co-occur in a single rat pup, therefore the functional link between these phenomena must remain speculative.

It should also be emphasized that although the first evidence of hippocampal function emerges at three weeks, fully adult-like spatial learning abilities do not emerge until considerably later [102,111,115,117,118,120,123,124]. Some of this protracted development could reflect the slow maturation of the visual system [53], and therefore the increasing ability to use distal visual cues [102,111,115]. However, the slow maturation of phenomena such as long-term retention of place memories [117,118], spontaneous alternation across a 30-s delay [124] or cross-modal redundancy in spatial processing [111] suggest that hippocampal function itself is still developing over this period, and this protracted maturation may be a reflection of the protracted development of place cells. The late development of long-term spatial memory [116,117,125] is particularly relevant to the observation that place cells in weanling rats have place fields which are relatively unstable over time (unlike those recorded from adult rats), suggesting that the inability of the hippocampus to form a stable map might underlie the long-term memory deficits observed in weanling and adolescent rats. An additional open question is whether the spatial memory deficits in immature rats, either at the behavioural level, or at the level of place field instability, map onto the phenomenon of infantile amnesia observed in humans [152] (see also [35] for work investigating the ontogeny of associative memory using non-spatial tasks).

Adult-like HD cells emerge very early, before most spontaneously expressed spatial behaviours [69,70,75]. It is therefore tempting to speculate that HD cell signalling may underlie some of the very earliest spatial behaviours observed in immature rats, such as taking a direct return path to the nest using path integration [71] or solving a visually cued water maze [107]. The possible causal link between HD cell development and homing using path integration is especially intriguing given recent evidence showing a correlation between HD cell accuracy and performance on a path integration-based homing task in adults [30]. Accurate HD cell signalling may therefore be a prerequisite in order for pups to commence exploration and return successfully to their nest, at an age when the other spatial signals (place and grid cells) are still not available to the organism. The finding that water maze performance in P17 pups was disrupted by the displacement of the platform relative to the pool [107] was interpreted by the authors as evidence for the adoption of directional strategy by the pups. This interpretation would be in keeping with the early emergence of HD cells. However, we would also emphasize the role of the pool boundaries in defining the location of the platform (see above) and note that preliminary evidence suggests that boundary responsive cells are present by P17 [142,143]. The ability of P17 pups to solve this ‘directional’ version of the water maze may therefore rely on the precocious development of a spatial system based on both direction and the boundaries of the environment.

As previously noted, all such functional links must remain speculative at this stage, in the absence of studies which directly test these hypotheses (though see [122]). We hope that the work outlined in this review will stimulate further research aimed at understanding the ontogeny of spatial behaviour and spatial function.
